# Multidimensional Emotional Disorder Inventory: Reliability and Validity in a Turkish Community Sample

**DOI:** 10.3390/bs16101957

**Published:** 2026-10-21

**Authors:** Merve Demir-Budakçıgil, Cennet Şafak-Öztürk, Gökhan Aksu, Mehmet Şakiroğlu, Anthony Joseph Rosellini

**Affiliations:** 1 Department of Psychology, Aydın Adnan Menderes University, Aydin 09010, Türkiye; cennet.ozturk@adu.edu.tr (C.Ş.-Ö.); mehmet.sakiroglu@adu.edu.tr (M.Ş.); 2 Division of Measurement and Evaluation in Education, Department of Educational Sciences, Faculty of Education, Aydın Adnan Menderes University, Aydin 09010, Türkiye; gokhanaksu@adu.edu.tr; 3 Department of Epidemiology, Boston University School of Public Health, Boston, MA 02118, USA; ajrosell@bu.edu

**Keywords:** Multidimensional Emotional Disorder Inventory, transdiagnostic assessment, emotional disorders, dimensional classification, measurement invariance, Turkish adaptation

## Abstract

Categorical diagnoses often fail to capture the comorbidity and heterogeneity of emotional disorders, and the Multidimensional Emotional Disorder Inventory (MEDI) offers a transdiagnostic, dimensional alternative. This study examined the psychometric properties of a Turkish version of the MEDI. The instrument was adapted following standardized cross-cultural procedures, including forward–backward translation, quantitative expert review of content validity, and a bilingual pilot study of linguistic equivalence. A community sample (*N* = 1011) completed the Turkish MEDI together with convergent validity measures. The factor structure was examined in two random half-samples using exploratory structural equation modeling (ESEM, n = 505) and ESEM and confirmatory factor analysis (*n* = 506), and ESEM-based measurement invariance was tested across administration mode (online vs. paper-and-pencil) and gender. ESEM supported the original nine-factor structure in both half-samples. All nine subscales showed good internal consistency (Cronbach’s α and McDonald’s ω = 0.80 to 0.88), and ESEM-based invariance tests supported scalar invariance across gender and administration mode, with weaker support for administration mode. Each MEDI dimension correlated strongly with its target criterion, supporting convergent validity, and gender-stratified community reference data were established. The findings support the reliability and validity of the Turkish MEDI for assessing transdiagnostic dimensions in community samples. Its utility for clinical decision-making requires further validation in clinical samples.

## 1. Introduction

Emotional disorders (EDs), which include anxiety, depressive, somatic symptom, and stressor-related disorders, are among the most common psychiatric conditions globally. Recent epidemiological data indicate that hundreds of millions of people are affected, making EDs a major contributor to the global burden of disease and a leading cause of disability-adjusted life years (GBD 2019 Mental Disorders Collaborators, 2022). Traditionally, the assessment and diagnosis of these conditions have depended on categorical classification systems, primarily the *Diagnostic and Statistical Manual of Mental Disorders* (DSM; American Psychiatric Association, 2013) and the *International Classification of Diseases* (ICD; World Health Organization, 2019). Although these nosologies have provided a necessary common language for clinicians, they are increasingly criticized for a categorical-splitting approach that is scientifically inconsistent with the nature of psychopathology (Brown & Barlow, 2009; Tung et al., 2020).

The most significant limitation of the categorical approach is the pervasive issue of diagnostic comorbidity (Brown & Barlow, 2009). Epidemiological and clinical studies consistently demonstrate that comorbidity among EDs is the rule rather than the exception (Antony & Barlow, 2020). For instance, a substantial majority of patients diagnosed with major depression also meet criteria for a concurrent anxiety disorder (Kessler et al., 2005; Mergl et al., 2007). This high rate of overlap suggests that anxiety and depressive disorders may not be separate etiological entities, but instead share underlying biological and psychological vulnerabilities, such as neuroticism and emotion dysregulation, that manifest heterogeneously across people (Barlow et al., 2014; Bullis et al., 2019). Furthermore, categorical approaches impose arbitrary thresholds on naturally dimensional symptoms. This often leads to the loss of important clinical information regarding subthreshold symptoms, which can significantly hinder functioning and cause distress even when they do not fully meet diagnostic criteria (Kliem et al., 2014; Osma et al., 2021). Consequently, the field has shifted toward transdiagnostic and dimensional models, such as the Hierarchical Taxonomy of Psychopathology (HiTOP) and the Research Domain Criteria (RDoC), which conceptualize psychopathology as a continuum of severity across shared latent traits (Insel et al., 2010; Kotov et al., 2017).

To operationalize this paradigm shift within a clinical context, Brown and Barlow (2009) proposed a hybrid dimensional-categorical profile approach. This model posits that EDs are best understood through a hierarchical structure comprising shared higher-order temperaments and lower-order symptom phenotypes. At the higher level, Neurotic Temperament (proneness to negative affect) and Positive Temperament (proneness to positive affect) serve as core etiological factors (Brown, 2007). At the lower level, specific dimensions such as autonomic arousal or social-evaluation concerns determine the phenotypic expression of the disorder (Rosellini & Brown, 2019).

To facilitate the application of this model, Rosellini and Brown (2019) developed the Multidimensional Emotional Disorder Inventory (MEDI). The MEDI is a 49-item self-report instrument designed to efficiently assess nine empirically supported transdiagnostic dimensions: (1) Neurotic Temperament, (2) Positive Temperament, (3) Depressed Mood, (4) Autonomic Arousal, (5) Somatic Anxiety, (6) Social Anxiety, (7) Intrusive Cognitions, (8) Traumatic Re-experiencing, and (9) Avoidance. Unlike traditional batteries that require multiple disorder-specific scales, which typically total nearly 90 items to cover the same constructs, the MEDI captures this heterogeneity in a single, concise administration. By evaluating these dimensions on a continuum, the MEDI enables the identification of specific symptom profiles that cut across traditional diagnostic boundaries (Boettcher et al., 2020; Osma et al., 2023). Because the nine dimensions represent conceptually distinct, and in some cases inversely related, constructs (e.g., Positive Temperament is negatively associated with Depressed Mood), the MEDI is scored as a profile of nine dimension scores rather than as a single total score.

While the MEDI has demonstrated robust psychometric properties in American clinical samples (Rosellini & Brown, 2019) and has been successfully adapted for Spanish (Osma et al., 2021, 2023), European Portuguese (Pereira et al., 2025), German (Bartholdy et al., 2026), and Colombian (Zárate-Guerrero et al., 2024) populations, its validity in the Turkish context remains unexplored. To our knowledge, no valid and reliable Turkish instrument assesses emotional disorders within a comprehensive, dimensional, transdiagnostic framework. This gap makes it difficult to measure transdiagnostic dimensions in Türkiye and to evaluate the effects of transdiagnostic interventions such as the Unified Protocol on these dimensions (Barlow & Farchione, 2018). A Turkish MEDI would provide a brief, common metric for these dimensions and would allow Turkish data to contribute to the cross-cultural evaluation of the hybrid model. Validating the MEDI in Türkiye is needed both to test the cross-cultural generalizability of the Brown and Barlow (2009) model and to address specific local assessment gaps. Similar to many non-Western and Mediterranean cultures, Turkish idioms of distress that somatize psychological suffering can shape the phenomenology of distress (Kirmayer et al., 1993; Wright & Bufka, 2020). People in these contexts often express emotional distress through somatic complaints (e.g., fatigue, pain, gastrointestinal distress, rushes of sensation) rather than through overt cognitive complaints of sadness or worry. Traditional categorical systems often struggle to classify these presentations, leading to potential misdiagnosis or the overuse of somatic symptom disorder diagnoses when an underlying anxiety or depressive process is present (Kirmayer et al., 1993). The MEDI is well suited to address this cultural nuance because it structurally distinguishes Somatic Anxiety (worry about health) and Autonomic Arousal (physical sensations) from Depressed Mood and Intrusive Cognitions. This structural separation allows researchers to identify high levels of somatic distress without conflating them with medical conditions or ignoring co-occurring affective deficits. The MEDI offers a transdiagnostic, culturally sensitive alternative that captures the comorbidity and somatic expressions of distress prevalent in this population within a single instrument.

The goal of this study was to validate the MEDI in a community sample in Türkiye. While clinical validation is essential, validating dimensional measures in community samples is equally critical for prevention and early intervention (Osma et al., 2021). Transdiagnostic dimensions such as neurotic temperament and avoidance are not exclusive to clinical populations; they also exist on a continuum in the general population (Barlow et al., 2014; Brown & Barlow, 2009; Kotov et al., 2017). Establishing the factor structure and reliability of the MEDI in a community sample allows for the examination of these traits across the full spectrum of severity, including subclinical manifestations (Osma et al., 2021). Individuals with high scores on transdiagnostic vulnerabilities are at elevated risk for developing diagnosable EDs (Barlow et al., 2014), and a validated general-population tool may enable early detection of these at-risk individuals before they meet diagnostic thresholds.

The aim of the present study was to develop the Turkish version of the MEDI and assess its psychometric properties in a large community sample. Specifically, this study aimed to (1) examine the factor structure of the Turkish MEDI to determine whether the original nine-factor structure replicates in a non-Western community context; (2) evaluate the internal consistency of the MEDI subscales; (3) test measurement invariance across administration mode and gender; (4) assess convergent and discriminant validity against established measures of anxiety, depression, and personality; and (5) establish normative data.

## 2. Materials and Methods

### 2.1. Participants

The study sample consisted of 1011 adults recruited from the general community in Türkiye. Participants were recruited using both online and in-person methods across the 12 sampled provinces. Online participants (*n* = 590, 58.4%) were reached through a Google Forms (Google LLC, Mountain View, CA, USA) survey distributed via social media platforms and various communication channels. In-person participants (*n* = 421, 41.6%) were recruited at family health centers, a university campus, and public venues (e.g., shopping centers, parks, and cultural centers), which were selected to reach individuals across a range of ages, education levels, and socioeconomic backgrounds. No professional survey company was used, participation was voluntary, and no incentive was offered. Twelve individuals were screened out because they were younger than 18 years or lacked native-level Turkish literacy, leaving 1073 respondents. Two attention-check items were embedded in the questionnaire; the responses of the 62 respondents who answered these items incorrectly were examined in detail, and most showed invariant or patterned responding (e.g., marking the same value for all items). These respondents were excluded from all analyses, yielding the final sample of 1011 participants. Screening for careless or invalid responding is recommended before psychometric analyses because such responses attenuate correlations and can distort factor structures (Meade & Craig, 2012). The excluded protocols (5.8% of respondents) were judged invalid and were therefore not retained in the analytic data set. The excluded respondents did not differ significantly from the analytic sample in age (*M* = 30.85 vs. 33.44, *p* = 0.062, d = −0.23), gender, marital status, education, income, place of residence, administration mode, or psychiatric medication use (χ^2^ or Fisher’s exact tests, all *p* > 0.08, Cramér’s *V* ≤ 0.08). Participants ranged in age from 18 to 65 years (*M* = 33.44, *SD* = 11.61). The majority identified as female (56.8%, *n* = 574) and 42.7% as male (*n* = 432); five participants (0.5%) did not report their gender. Inclusion criteria required that participants be at least 18 years of age and possess native-level Turkish literacy to ensure full conceptual and linguistic understanding of the items.

To achieve broad geographical coverage, recruitment was organized according to the Nomenclature of Territorial Units for Statistics (NUTS) Level-1 regions. Türkiye is divided into 12 NUTS-1 regions; within each region, the most populous province was selected, and participants in these provinces were recruited through convenience (non-probability) methods. The sample therefore provides broad geographical coverage but is not statistically representative of the Turkish adult population. The target sample size was approximately 1000 participants, so that each of the two half-samples would include about 500 participants. The adequacy of this sample size was evaluated in two ways. First, an RMSEA-based power analysis (MacCallum et al., 1996) with α = 0.05 and the 771 degrees of freedom of the nine-factor ESEM showed that, with *n* = 505, power exceeded 0.99 both for the test of close fit (H0: RMSEA = 0.05 vs. H1: RMSEA = 0.08) and for the test of not-close fit (H0: RMSEA = 0.05 vs. H1: RMSEA = 0.01). Second, because such power analyses address only overall model fit, the precision of the parameter estimates was examined with a Monte Carlo simulation (Muthén & Muthén, 2002) with 1000 replications of *n* = 505, using the estimates of the nine-factor CFA in the confirmatory half-sample as population values. All replications converged to proper solutions. Parameter and standard error biases were below 10% for all parameters. Standard error bias exceeded 5% only slightly for two factor loadings (5.1% and 6.4%), one factor correlation (6.3%), and one residual variance (5.9%). Coverage of the 95% confidence intervals ranged from 0.916 to 0.963, and power was at least 0.96 for all factor loadings and factor correlations. These results are largely consistent with the criteria of Muthén and Muthén (2002). Because the simulation assumed normally distributed continuous indicators, these results should be regarded as approximate. Completing the full questionnaire set took approximately 15–20 min. Detailed sociodemographic characteristics of the sample are presented in Table 1.

### 2.2. Measures

#### 2.2.1. Multidimensional Emotional Disorder Inventory (MEDI)

The MEDI is a 49-item transdiagnostic self-report inventory developed by Rosellini and Brown (2019) to efficiently assess the nine dimensions of the hybrid dimensional-categorical profile approach: two higher-order temperamental factors (neurotic and positive temperament) and seven lower-order phenotypic dimensions (depressed mood, autonomic arousal, somatic anxiety, social anxiety, intrusive cognitions, traumatic re-experiencing, and avoidance). Items are rated on a 9-point Likert-type scale ranging from 0 (“Not characteristic of me/Does not apply to me”) to 8 (“Extremely characteristic of me/Applies to me very much”). In the original development study, the MEDI demonstrated a robust nine-factor structure and acceptable composite reliability (*ρ* = 0.68 to 0.93) across all dimensions.

#### 2.2.2. Big Five Inventory (BFI)

The BFI (Benet-Martínez & John, 1998; Turkish adaptation by Sümer & Sümer, 2005) assesses the five-factor model of personality. The Neuroticism and Extraversion subscales were used to establish convergent validity for the MEDI Neurotic Temperament and Positive Temperament scales. In the present sample, internal consistency was α = 0.86 (Neuroticism) and α = 0.83 (Extraversion).

#### 2.2.3. Depression Anxiety Stress Scales-21 (DASS-21)

The DASS-21 assesses symptoms of depression, anxiety, and stress over the past week; the Turkish adaptation was conducted by Sarıçam (2018). The Depression and Anxiety subscales were used to validate the MEDI Depressed Mood and Autonomic Arousal dimensions. In the present sample, α = 0.87 (Depression) and α = 0.86 (Anxiety).

#### 2.2.4. Brief Fear of Negative Evaluation Scale (BFNE)

The BFNE (Leary, 1983; Turkish adaptation by Çetin et al., 2010) measures apprehension about others’ evaluations and was used to assess the convergent validity of the MEDI Social Anxiety dimension. In the present sample, α = 0.81.

#### 2.2.5. Obsessive-Compulsive Inventory-Revised (OCI-R)

The OCI-R (Foa et al., 2002; Turkish adaptation by Yorulmaz et al., 2015) assesses six symptom domains of obsessive–compulsive disorder. The Obsessing subscale was used to validate the MEDI Intrusive Cognitions dimension. In the present sample, α = 0.80.

#### 2.2.6. Anxiety Sensitivity Index-3 (ASI-3)

The ASI-3 (Taylor et al., 2007; Turkish adaptation by Mantar et al., 2010) assesses the fear of anxiety-related sensations. The Physical Concerns subscale was used to validate the MEDI Somatic Anxiety dimension. In the present sample, α = 0.84.

#### 2.2.7. PTSD Checklist for DSM-5 (PCL-5)

The PCL-5 (Blevins et al., 2015; Turkish adaptation by Boysan et al., 2017) assesses PTSD symptoms according to DSM-5 criteria. The Re-experiencing subscale was used to establish convergent validity for the MEDI Traumatic Re-experiencing dimension. In the present sample, α = 0.77.

#### 2.2.8. Multidimensional Experiential Avoidance Questionnaire (MEAQ)

The MEAQ (Gámez et al., 2011; Turkish adaptation by Yıldırım & Bahtiyar, 2020) assesses avoidance strategies and was used to validate the MEDI Avoidance dimension. In the present sample, α = 0.92.

### 2.3. Procedure

The adaptation of the MEDI followed the ten-step translation and cultural-adaptation process recommended by the ISPOR Task Force for Translation and Cultural Adaptation (Wild et al., 2005), a widely used standard for adapting self-report measures (Beaton et al., 2000; International Test Commission, 2017). First, permission was obtained from the original developers, who also provided clarifying information about the scale’s constructs for use during translation. A team of five independent translators, all native Turkish speakers holding doctoral degrees in psychology and familiar with the constructs assessed, independently conducted the forward translation of the 49 items, prioritizing conceptual equivalence and language readily understood by the general population over literal translation. These translations were reconciled into a single Turkish form, which was back-translated into English by five different independent translators who had not taken part in the forward translation. The back-translated items were systematically compared with the original English items to identify conceptual and linguistic discrepancies, and the Turkish form was revised accordingly; translation decisions were then harmonized with previously published adaptations of the scale.

As part of the cognitive-debriefing step, the resulting Turkish form was reviewed by two clinical psychology professors for cultural appropriateness and conceptual equivalence, and revisions were made in line with their feedback. Content validity was then quantified by a separate panel of five expert psychologists, each of whom independently rated all 49 items on a 3-point scale (1 = should be removed, 2 = should be revised, 3 = appropriate). For each item, the Content Validity Ratio (CVR) was computed with Lawshe’s (1975) formula, CVR = (*n*_e_ − *N*/2)/(*N*/2), where *n*_e_ is the number of experts rating the item as appropriate and *N* is the total number of experts. The Content Validity Index (CVI) was computed as the mean CVR across items (Veneziano & Hooper, 1997). With five experts, the critical CVR at α = 0.05 (one-tailed) is 1.00 (Ayre & Scally, 2014), corresponding to the value of 0.99 in Lawshe’s (1975) original table, so an item was accepted without revision only when all five experts rated it as appropriate. In addition, inter-rater agreement among the five experts was assessed with Gwet’s AC1 (Gwet, 2002). Items falling below the critical threshold were revised in line with the experts’ feedback and re-evaluated.

To assess linguistic equivalence, a pilot study was conducted with 45 bilingual undergraduate students enrolled in the Department of English Language and Literature at Aydın Adnan Menderes University (*M* age = 22.60 years, *SD* = 1.76, 56% men), who completed both the English and Turkish versions in a single session, with the original English form administered first. The sample size was determined by the number of eligible bilingual students who volunteered. With *n* = 45, the equivalence test described below had a power of approximately 0.90 to establish equivalence within bounds of ±0.5 standard deviations of the difference scores when the true difference was zero. Because no established equivalence bounds exist for this type of comparison, the bounds were set in line with Lakens’ (2017) recommendation to base them on the effect sizes that the available sample can detect with adequate power. Equivalence was evaluated with paired-samples *t* tests, Pearson correlations, two one-sided tests (TOST) for equivalence (Lakens, 2017), and two-way random-effects intraclass correlation coefficients for absolute agreement [ICC(A,1); McGraw & Wong, 1996]. Items showing statistically significant differences between the two forms were re-examined with the translators and revised. Data collection for the main study was voluntary. Before participation, all participants were informed about the aim of the study, the voluntary nature of participation, their right to withdraw at any time, and the confidentiality of their data. Participants in the paper-and-pencil condition signed a written informed consent form, and participants in the online condition provided electronic informed consent on the first page of the survey before accessing the questionnaires. The study was approved by the Social and Human Sciences Research Ethics Committee of Aydın Adnan Menderes University (decision no. 11/10, 9 February 2024).

### 2.4. Data Analysis

Analyses were conducted in SPSS 26.0 (IBM Corp., Armonk, NY, USA; IBM Corp., 2019) and R version 4.6.1 (R Foundation for Statistical Computing, Vienna, Austria) with the lavaan package (version 0.7-2; Rosseel, 2012). Missing data were minimal (0.04% of item responses) and were handled with full-information maximum likelihood estimation. The five participants who did not report their gender were excluded from the gender-based invariance analysis. Normality was assessed via skewness, kurtosis and standardized *z*-scores.

To examine the internal structure while minimizing the risk of overfitting from analyzing the same data twice, the total sample was randomly split into two independent half-samples. The split was stratified by administration mode and carried out in R with a fixed random seed (20261014), so that each half-sample contained 295 online participants and 210 or 211 paper-and-pencil participants. The half-samples were compared on sociodemographic characteristics with χ^2^ tests and a Welch *t* test (Supplementary Table S1). In the first half-sample (*n* = 505), the structure was examined with exploratory structural equation modeling (ESEM), estimated with robust maximum likelihood (MLR) and quartimin rotation, consistent with the original development study (Rosellini & Brown, 2019). The ESEM was specified with a fully saturated loading matrix, in which all items were free to load on all factors, and no correlated residuals, making it equivalent to an exploratory factor analysis (EFA) while additionally providing model fit indices and modification indices. ESEM was preferred over a conventional EFA because it estimates the same freely rotated measurement model within a structural equation modeling framework, thereby providing standard errors, goodness-of-fit indices, and the possibility of testing measurement invariance with the same model that is used to examine the factor structure (Asparouhov & Muthén, 2009; Marsh et al., 2014). In the second half-sample (*n* = 506), the nine-factor structure was cross-validated with both an ESEM and a confirmatory factor analysis (CFA). The ESEM served as the primary structural framework, following the original study, whereas the CFA provided a complementary, strictly confirmatory test comparable to prior MEDI adaptations. Model fit was evaluated using the model chi-square (χ^2^), the χ^2^/*df* ratio, the root mean square error of approximation (RMSEA), the comparative fit index (CFI), the Tucker-Lewis index (TLI), the normed fit index (NFI), the incremental fit index (IFI), and the standardized root mean square residual (SRMR). For the ESEM models, which were estimated with MLR, the scaled χ^2^ and the robust CFI, TLI, and RMSEA are reported. The CFA models were estimated with maximum likelihood, and nested CFA models were compared with the χ^2^ difference test, the Akaike information criterion (AIC), and the expected cross-validation index (ECVI). Established cut-offs were used to determine acceptable fit, with χ^2^/*df* of 3 or below, CFI and TLI of 0.95 or above, and RMSEA and SRMR of 0.08 or below (Hu & Bentler, 1999). Accordingly, the ESEM was treated as the primary test of the internal structure, and the CFA as a complementary test. All factor models were estimated in R (lavaan), and no correlated residuals or other post hoc modifications were added.

Internal consistency was evaluated for each of the nine subscales using Cronbach’s alpha and McDonald’s omega (each computed from a single-factor model of the respective subscale’s items); consistent with the profile-based scoring of the MEDI, no overall/total-score reliability was computed.

Measurement invariance was tested in the full sample in R (lavaan), separately for administration mode (online vs. paper-and-pencil) and gender (female vs. male), by sequentially comparing configural, metric (equal loadings), and scalar (equal loadings and intercepts) models. Because the nine-factor CFA showed only modest configural fit, invariance was tested primarily within the ESEM framework (multi-group ESEM with MLR estimation and quartimin rotation), which does not constrain cross-loadings to zero (Marsh et al., 2014). Multi-group CFA models (maximum likelihood) are also reported for comparability with previous MEDI adaptations. A decrease in CFI of 0.010 or more was considered the main indicator of noninvariance (Cheung & Rensvold, 2002). Following Chen (2007), noninvariance was inferred when this decrease was supplemented by an increase in RMSEA of 0.015 or more or an increase in SRMR of 0.030 or more for loading invariance (0.010 or more for intercept invariance). Because ESEM invariance models differ greatly in the number of estimated parameters, the parsimony-corrected TLI and RMSEA were also considered. Following Marsh et al. (2010), the more constrained model was regarded as supported when its TLI or RMSEA was as good as or better than that of the less constrained model. Convergent and discriminant validity were examined by computing summed scale scores for each MEDI subscale and each criterion measure and then estimating Pearson correlations among them. Discriminant validity was further evaluated with the latent factor intercorrelations estimated by the ESEM in the confirmatory half-sample.

## 3. Results

### 3.1. Content and Language Validity

Content validity was quantified from the ratings of five expert psychologists, each of whom rated all 49 items on a 3-point scale (1 = should be removed, 2 = should be revised, 3 = appropriate). In the first round, the Content Validity Index, computed as the mean Content Validity Ratio (Ayre & Scally, 2014) across items, was 0.94; seven items (items 1, 3, 6, 10, 13, 25, and 26) fell below the critical value and were revised in line with the experts’ feedback. In the second round, all item Content Validity Ratios and the overall Content Validity Index reached 1.00. Inter-rater agreement among the five experts was almost perfect (Gwet’s AC1 = 0.95, *SE* = 0.02, 95% CI [0.91, 0.99]; Landis & Koch, 1977).

Language equivalence was assessed in the bilingual sample (*N* = 45) who completed both the Turkish and English forms. Paired-samples *t* tests showed no statistically significant difference between the Turkish and English forms on any of the nine subscales (all *p* ≥ 0.21), and subscale scores were highly correlated across forms (Pearson *r* = 0.84 to 0.98, all *p* < 0.001). Because a non-significant difference does not by itself demonstrate equivalence, TOST equivalence tests were conducted with bounds of ±0.5 standard deviations of the difference scores. Equivalence was supported for all nine subscales (all pTOST ≤ 0.022). In raw score units, the equivalence bounds ranged from ±0.99 to ±2.49 points, which corresponds to at most 0.28 pooled standard deviations of the subscale scores. ICC(A,1) point estimates ranged from 0.84 to 0.99, indicating good to excellent absolute agreement, although the 95% CI for Positive Temperament extended into the moderate range (lower bound = 0.73, Koo & Li, 2016). Full results are given in Supplementary Table S3. At the item level, 45 of the 49 items showed no significant difference between the forms. Four items differed at *p* < 0.05: items 16 (neurotic temperament), 38 (somatic anxiety), and 42 (avoidance) scored lower on the Turkish form, and item 47 (social anxiety) scored higher, with mean differences of 0.20 to 0.42 scale points. Given 49 comparisons, two to three significant differences would be expected by chance. Nevertheless, these items were reviewed with the translators and revised before the final Turkish form was established.

### 3.2. Preliminary Analyses

Little’s test (Little, 1988) indicated that the data were missing completely at random, χ^2^(2150) = 1945.80, *p* = 0.99. Missing data were minimal, affecting 19 item responses (0.04%) from 18 participants. Missing values were handled with full-information maximum likelihood (FIML) estimation. Descriptive statistics for the nine subscales are presented in Table 2. Subscale skewness values ranged from −0.60 to 0.51 and kurtosis from −0.84 to −0.28, all within ±1.5, and no standardized *z*-score exceeded ±3.29, supporting the assumption of univariate normality. Item-level response distributions are provided in Supplementary Table S2.

The two half-samples did not differ in age, gender, marital status, education, or income (all *p* ≥ 0.15, Cramér’s *V* ≤ 0.08). A small difference was found for place of residence (*p* = 0.018, *V* = 0.10). Detailed comparisons are given in Supplementary Table S1.

### 3.3. Construct Validity: Factor Structure

The internal structure was examined using ESEM in the first half-sample (*n* = 505) and cross-validated in the second half-sample (*n* = 506), where both an ESEM and a confirmatory factor analysis (CFA) were estimated. Following the original development study (Rosellini & Brown, 2019), all ESEM models used robust maximum likelihood estimation and quartimin rotation. In the first half-sample, sampling adequacy was excellent (Kaiser–Meyer–Olkin = 0.95), and Bartlett’s test of sphericity was significant, χ^2^(1176) = 14,435.13, *p* < 0.001, confirming the suitability of the data for factor analysis.

The exploratory ESEM fit the data adequately, χ^2^(771) = 1555.40, χ^2^/*df* = 2.02, RMSEA = 0.046 (90% CI [0.042, 0.050]), SRMR = 0.024, CFI = 0.95, TLI = 0.93. Table 3 presents the complete loading matrix. The hypothesized nine-factor structure was largely recovered, with 43 of the 49 items showing their primary loading on the intended dimension. The main exception concerned the distinction between Autonomic Arousal and Somatic Anxiety. Three Autonomic Arousal items (4, 26, and 44) loaded most strongly on Somatic Anxiety, and the Somatic Anxiety item 6 loaded slightly more strongly on Neurotic Temperament (0.39) than on its target factor (0.34). In addition, two Avoidance items (23 and 49) loaded primarily on the Autonomic Arousal factor. Nine cross-loadings reached 0.30 or higher. The exploratory solution should therefore be interpreted with caution, and these items were examined again in the confirmatory half-sample.

The nine-factor structure was then cross-validated in the second half-sample. The ESEM again fit the data adequately, χ^2^(771) = 1532.60, χ^2^/*df* = 1.99, RMSEA = 0.046 (90% CI [0.041, 0.050]), SRMR = 0.024, CFI = 0.95, TLI = 0.92. Every item loaded most strongly on its intended dimension, target loadings ranged from 0.34 to 0.90, and no cross-loading reached 0.30 (Table 4). The a priori nine-factor CFA, estimated without correlated residuals or other modifications, fit only modestly, χ^2^(1091) = 3345.68, χ^2^/*df* = 3.07, RMSEA = 0.064 (90% CI [0.061, 0.066]), SRMR = 0.068, CFI = 0.82, TLI = 0.81. It nevertheless fit significantly better than an eight-factor model in which Traumatic Re-experiencing and Intrusive Cognitions were merged, χ^2^(1099) = 3745.49, RMSEA = 0.069, CFI = 0.79, Δχ^2^(8) = 399.81, *p* < 0.001, and it had lower AIC (105,554.94 vs. 105,938.75) and ECVI (7.34 vs. 8.09) values. The latent factor intercorrelations were considerably lower under ESEM than under CFA (highest *r* = 0.52 vs. 0.78, mean |*r*| = 0.27 vs. 0.55). This pattern indicates that the weaker fit and the higher factor correlations of the CFA largely reflect the requirement that all cross-loadings be exactly zero (Marsh et al., 2014). For example, item 38 had the weakest Somatic Anxiety loading in the CFA (λ = 0.38) but loaded adequately on its target factor under ESEM (λ = 0.63). The results from both half-samples therefore support a nine-factor structure for the Turkish MEDI, with ESEM representing the data better than CFA.

### 3.4. Reliability

Internal consistency was evaluated for each of the nine subscales; consistent with the profile-based scoring of the MEDI, no total-score reliability was computed. As shown in Table 4, Cronbach’s α ranged from 0.80 to 0.88 and McDonald’s ω from 0.80 to 0.88: neurotic temperament (α = 0.85, ω = 0.85), positive temperament (0.81, 0.82), depressed mood (0.81, 0.82), autonomic arousal (0.87, 0.87), somatic anxiety (0.80, 0.80), social anxiety (0.86, 0.86), intrusive cognitions (0.88, 0.88), traumatic re-experiencing (0.85, 0.86), and avoidance (0.83, 0.83). All subscales thus exceeded the conventional 0.70 threshold.

### 3.5. Measurement Invariance

Measurement invariance was first examined with multi-group CFA (Table 5 and Table 6), as in previous MEDI adaptations. The configural CFA models fit only modestly (CFI = 0.821, RMSEA = 0.066, and SRMR = 0.072 for administration mode, and CFI = 0.835, RMSEA = 0.063, and SRMR = 0.067 for gender), which limits the interpretability of CFA-based invariance tests. Invariance was therefore tested primarily with multi-group ESEM, which yielded substantially better configural fit (robust CFI = 0.941, TLI = 0.910, RMSEA = 0.050, and SRMR = 0.025 for administration mode, and CFI = 0.949, TLI = 0.923, RMSEA = 0.047, and SRMR = 0.024 for gender). For gender (*n* = 1006, Table 6), CFI decreased by 0.011 when the loadings were constrained, but RMSEA decreased (0.047 to 0.043) and ΔSRMR (0.013) remained below 0.030, so the combined criteria for noninvariance (Chen, 2007) were not met. TLI also improved (0.923 to 0.924). Additionally, constraining the intercepts produced no further change (ΔCFI = 0.000), supporting scalar invariance across gender. For administration mode (Table 5), CFI decreased by 0.021 at the metric level, which exceeded the 0.01 criterion, but RMSEA did not increase (0.050 to 0.049) and ΔSRMR (0.014) remained below 0.030, so the combined criteria for noninvariance were not met. TLI decreased slightly (0.910 to 0.901). Additionally, constraining the intercepts produced no meaningful further change (ΔCFI = −0.001). According to these criteria and to the guideline that a more parsimonious model is supported when its TLI or RMSEA is as good as or better than that of the less constrained model (Marsh et al., 2010), the results support metric and scalar invariance across administration mode, although the change in CFI alone exceeded 0.01 and such guidelines should be regarded as rules of thumb. In the CFA models, all changes in CFI were ≤0.006 for both grouping variables (Table 5 and Table 6), although these tests must be interpreted in light of the modest configural fit. Taken together, the findings support measurement invariance across gender and administration mode, with somewhat weaker evidence for administration mode.

### 3.6. Convergent and Discriminant Validity

Convergent and discriminant validity were assessed via Pearson correlations between the MEDI subscales and the criterion measures (Table 7). All convergent correlations were statistically significant (*p* < 0.001) and in the expected direction. Moreover, each convergent correlation exceeded the correlations of the corresponding criterion measure with the other MEDI subscales reported in Table 7, providing preliminary support for discriminant validity. Further evidence of discriminant validity came from the ESEM factor intercorrelations in the confirmatory half-sample, which were low to moderate (mean |*r*| = 0.27, highest *r* = 0.52), indicating that the nine dimensions share variance but are empirically distinguishable. Neurotic Temperament correlated with BFI-Neuroticism (*r* = 0.86) and Positive Temperament with BFI-Extraversion (*r* = 0.83). Among the phenotype dimensions, Depressed Mood correlated with DASS-Depression (*r* = 0.75), Autonomic Arousal with DASS-Anxiety (*r* = 0.81), Intrusive Cognitions with the OCI-R Obsessing subscale (*r* = 0.79), Traumatic Re-experiencing with the PCL-5 Re-experiencing subscale (*r* = 0.83), and Avoidance with the MEAQ (*r* = 0.81). Social Anxiety correlated with the BFNE (*r* = 0.58) and Somatic Anxiety with the ASI-3 Physical Concerns subscale (*r* = 0.54).

### 3.7. Normative Data

**Figure 1:**
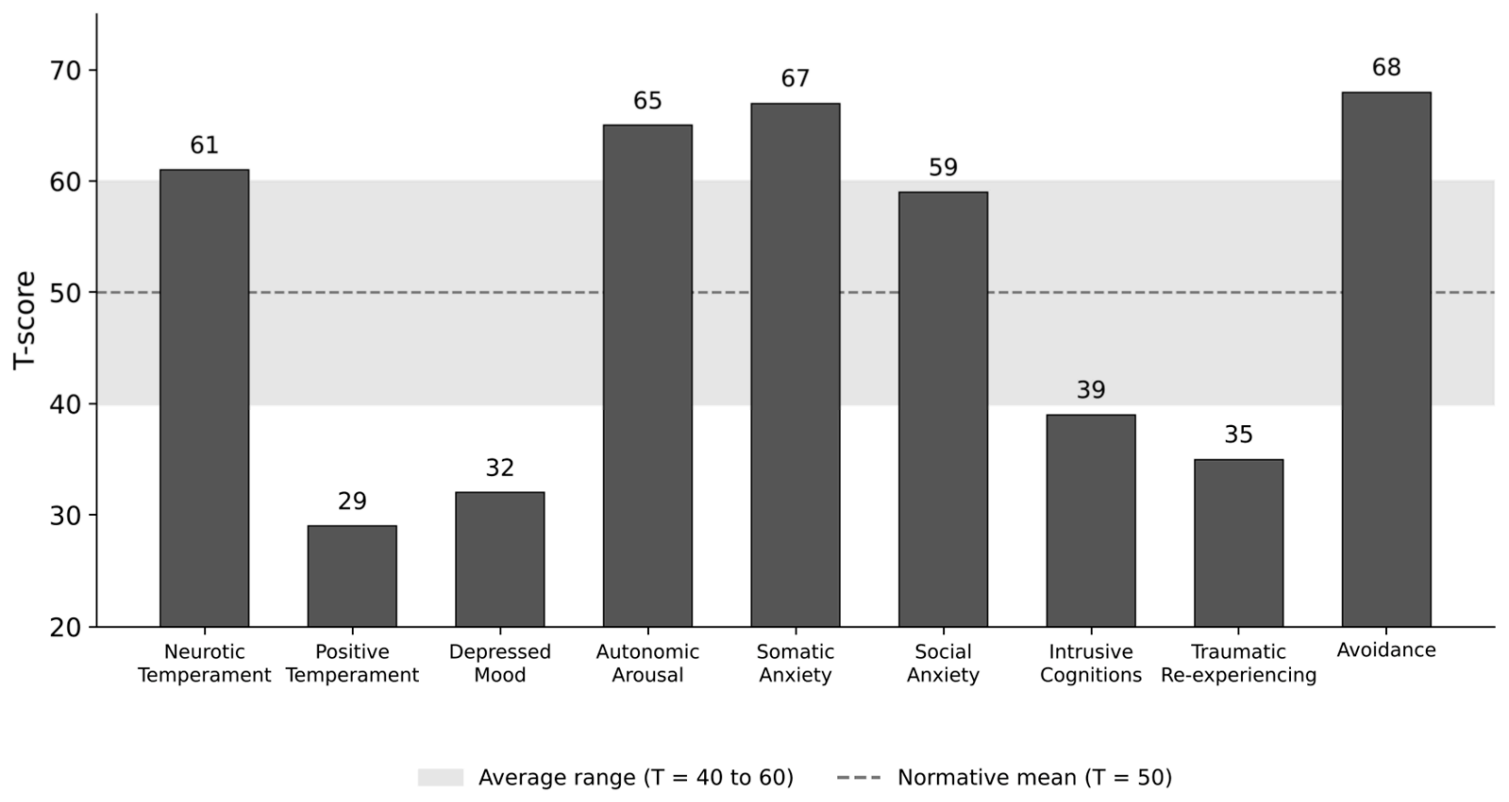
Illustrative MEDI profile for a single respondent. *T*-scores are standardized within the respondent’s gender group (*M* = 50, *SD* = 10). The shaded band denotes the average range (*T* = 40 to 60), and the dashed line denotes the normative mean (*T* = 50). MEDI = Multidimensional Emotional Disorder Inventory.

Gender-specific normative data were established to facilitate the interpretation of individual scores relative to the community reference group. As presented in Table 8, percentile ranks and standardized *T*-scores were computed within each gender group for all nine dimensions, allowing clinicians to situate an individual’s transdiagnostic profile within the general-population distribution. Because the sample was not drawn by probability sampling, these values should be regarded as community-based reference data rather than nationally representative norms. To illustrate this profile-based interpretation, Figure 1 presents the standardized profile of a single respondent from the sample. This individual showed marked elevations on Avoidance (*T* = 68), Somatic Anxiety (*T* = 67), and Autonomic Arousal (*T* = 65), together with below-average scores on Depressed Mood (*T* = 32), Intrusive Cognitions (*T* = 39), and Traumatic Re-experiencing (*T* = 35). This pattern illustrates the capacity of the Turkish MEDI to distinguish a predominantly somatic and avoidance-based presentation from depressive and trauma-related symptomatology, information that a single categorical diagnosis would obscure.

## 4. Discussion

The present study evaluated the psychometric properties of the Turkish adaptation of the MEDI in a large community sample. The results provide support for the reliability and validity of the Turkish MEDI as a transdiagnostic instrument that captures meaningful variation in emotional-disorder liability across both higher-order temperamental vulnerabilities and lower-order symptom phenotypes. Beyond linguistic adaptation, this validation facilitates a transition from traditional categorical frameworks toward a hybrid dimensional-categorical profile approach within the Turkish context (Brown & Barlow, 2009; Rosellini & Brown, 2019). At the same time, several findings call for caution. In the exploratory half-sample, six items did not show their primary loading on the intended dimension and several items cross-loaded. In addition, eight target loadings in the exploratory half-sample and four in the confirmatory half-sample were below 0.40, and the CFA models fit only modestly.

Consistent with the original development study and validations in Spain and Germany, the ESEM supported the nine-factor model, and in the complementary CFA the nine-factor model clearly outperformed a more parsimonious eight-factor alternative (Rosellini & Brown, 2019; Osma et al., 2023; Bartholdy et al., 2026). In both half-samples, all Traumatic Re-experiencing items loaded on their intended dimension. This separation is noteworthy given the phenomenological overlap between intrusive obsessive thoughts and intrusive trauma memories (Rosellini et al., 2015). While a recent Colombian study found an eight-factor model to be the best-fitting solution (Zárate-Guerrero et al., 2024), our competing-models approach confirmed the superiority of the nine-factor structure, suggesting that the Turkish adaptation successfully distinguishes traumatic from general cognitive intrusions. Preserving this distinction is clinically important, because autobiographical trauma requires different processing techniques than the nonsensical intrusions typical of obsessive-compulsive disorder (Brewin et al., 2010; Rosellini & Brown, 2019).

Several findings replicate previous MEDI validations, whereas others appear specific to the Turkish data. Support for the nine-factor structure, the good internal consistency of the subscales, and the pattern of convergent correlations with established criterion measures are consistent with the original study and with the Spanish, German, and Portuguese validations (Rosellini & Brown, 2019; Osma et al., 2021, 2023; Bartholdy et al., 2026; Pereira et al., 2025). By contrast, three Autonomic Arousal items (4, 26, and 44) showed their strongest loadings on Somatic Anxiety in the exploratory half-sample, and Somatic Anxiety showed the weakest convergent correlation in the study (*r* = 0.54 with ASI-3 Physical Concerns). Research with Turkish patients with depression indicates that the correlates of somatic symptom attribution differ from those reported in Western countries, possibly reflecting cultural characteristics and help-seeking pathways (Taycan et al., 2015), and that somatic symptom reporting is closely linked to difficulty in identifying emotions (Taycan et al., 2017). Such characteristics may contribute to a less sharp differentiation between bodily sensations and health concerns in the Turkish data. Because this pattern was not replicated in the confirmatory half-sample, it should be examined in clinical Turkish samples before it is interpreted as a cultural characteristic.

No correlated residuals were added to the CFA, so that the reported model would not be tailored to the present sample. Under this specification, the CFA fit only modestly, whereas the ESEM fit adequately without any modification and yielded markedly lower factor intercorrelations. This contrast indicates that the strict independent-clusters structure of CFA, rather than the nine-factor structure itself, accounts for most of the misfit, which is consistent with the known limitations of CFA for multidimensional item-level measures, namely poor fit and inflated factor correlations (Marsh et al., 2014). Item 38 (health vigilance) had the weakest Somatic Anxiety loading in the CFA (λ = 0.38), although it loaded adequately under ESEM. In the European Portuguese validation and the Spanish adolescent validation, the residuals of this item and item 19 (health worry) were correlated (Pereira et al., 2025; Pérez-Esteban et al., 2024), which suggests that health vigilance shares variance with health worry beyond the Somatic Anxiety factor. The functioning of item 38 therefore warrants attention in future revisions.

A further contribution of this study is the examination of measurement invariance across administration mode and gender, an analysis rarely reported in previous MEDI adaptations. ESEM-based tests supported scalar invariance across gender, converging with the German validation (Bartholdy et al., 2026) and indicating that observed gender differences are unlikely to reflect measurement artifacts, which provides a basis for the gender-specific reference data reported here. Invariance across online and paper-and-pencil administration, to our knowledge tested here for the first time for the MEDI, was also supported: when the loadings were constrained, RMSEA did not increase, and SRMR changed only slightly, although the decrease in CFI exceeded the conventional criterion (Chen, 2007; Marsh et al., 2010). Pooling data across administration modes therefore appears justified, but confirmation in independent samples, ideally with participants randomly assigned to administration mode, is desirable. In addition, the modest fit of the configural CFA models indicates that the strict independent-clusters structure assumed by CFA does not fully capture the Turkish data, and that the ESEM framework, which allows small cross-loadings, provides a more appropriate basis for invariance testing with the MEDI.

The identification of higher-order temperamental vulnerabilities is central to this transdiagnostic approach. Consistent with the General Neurotic Syndrome framework, the Neurotic Temperament subscale appears to represent a core factor in the development and maintenance of emotional disorders in the Turkish context (Andrews, 1996; Barlow et al., 2014); its robust correlation with negative affectivity is consistent with the conceptualization of neuroticism as a fundamental diathesis for internalizing disorders (Sauer-Zavala, 2022). The negative correlation between Positive Temperament and Depressed Mood is consistent with the tripartite model, which identifies low positive affect as a specific marker of depression (Clark & Watson, 1991). The strong pattern of convergent correlations further supports the interpretability of the nine dimensions as distinct but related constructs. The very high correlation between Neurotic Temperament and BFI-Neuroticism (*r* = 0.86) is consistent with the definition of Neurotic Temperament as the trait tendency to experience negative affect, which includes neuroticism, and with the strong latent correlation between Neurotic Temperament and NEO-FFI Neuroticism in the original study (φ = 0.79; Rosellini & Brown, 2019). At the same time, it indicates that the two scales are largely interchangeable as measures of this temperamental vulnerability, and the added value of the MEDI lies mainly in placing it within the same profile as the seven phenotypic dimensions. More generally, the MEDI dimensions were moderately intercorrelated. Although each convergent correlation exceeded the corresponding cross-construct correlations and the ESEM factor intercorrelations were at most moderate, the discriminant validity of closely related dimensions (e.g., Autonomic Arousal and Somatic Anxiety, or Intrusive Cognitions and Traumatic Re-experiencing) should be examined further, for example with multitrait–multimethod designs.

A potential clinical application of the MEDI is to inform mechanism-based interventions such as the Unified Protocol (UP). In contrast to tools that yield categorical labels, the MEDI provides data that may inform treatment planning (Boettcher et al., 2020). For example, elevated Autonomic Arousal may indicate the relevance of the UP module on awareness and tolerance of physical sensations and the use of interoceptive exposure, irrespective of whether the individual meets criteria for panic disorder or somatic symptom disorder (Wilamowska et al., 2010); elevated Avoidance may guide clinicians toward the emotion-driven-behaviors module (Anderson et al., 2021; Barlow & Farchione, 2018). A profile marked by elevated Depressed Mood and low Positive Temperament may indicate a greater need for behavioral activation (Rosellini et al., 2015). This profile-based approach may address limitations of single-disorder protocols, which frequently do not account for the heterogeneous presentation of comorbid symptoms (Steele et al., 2018). Because the present study used a community sample and did not evaluate treatment outcomes or clinical decision-making, these applications remain hypotheses that require validation in clinical samples.

The findings should be interpreted in light of several limitations. First, the validation used a community-based sample recruited through non-probability methods. Although recruitment covered all 12 NUTS-1 regions, only one province per region was sampled, and most participants (59.8%) lived in metropolitan cities. The sample is therefore not representative of the Turkish population, and the reference data in Table 8 should be regarded as community-based rather than national norms. Replication in clinical samples is also needed to establish generalizability to treatment-seeking populations (Osma et al., 2023). Second, reliance on self-report introduces potential response biases; future research should incorporate structured clinical interviews, which would also allow the diagnostic accuracy of MEDI scores (e.g., sensitivity and specificity for specific disorders) to be evaluated. Third, the cross-sectional design precluded assessment of test-retest reliability and sensitivity to change; longitudinal studies are required to determine the utility of the Turkish MEDI in tracking symptom change during transdiagnostic interventions. Fourth, the configural CFA models fit only modestly, and at the metric level the decrease in CFI exceeded 0.01 for administration mode (and marginally for gender), although the combined criteria for noninvariance were not met. Because administration mode was also confounded with sociodemographic characteristics in this sample, invariance across modes should be confirmed in independent samples. Finally, language equivalence was examined in a small, highly educated sample of bilingual students who completed both forms in a single session in a fixed order (English first). Memory and order effects may therefore have increased the agreement between the forms, and the equivalence findings may not generalize to other populations.

## 5. Conclusions

The validation of the Turkish MEDI represents a meaningful advance in the assessment of emotional disorders, facilitating a transition from taxonomic theory to clinical application. By supporting the nine-factor structure, providing evidence of measurement invariance across gender and administration mode, and demonstrating good reliability and convergent validity, this study provides an assessment tool that addresses key limitations of categorical diagnostic systems. Additionally, the MEDI enables practitioners to address the comorbidity paradox by focusing on shared underlying mechanisms, which may facilitate the implementation of transdiagnostic interventions such as the UP. Ultimately, this research underscores the importance of precise dimensional measurement for understanding the processes underlying psychological distress, and the Turkish MEDI may support research and, once validated in clinical samples, treatment planning within the Turkish context.

## Figures and Tables

**Table 1:** Sociodemographic characteristics of the sample (*N* = 1011).

Characteristic	*n*	%
Administration mode		
Online	590	58.4
Paper-and-pencil	421	41.6
Gender		
Female	574	56.8
Male	432	42.7
Not reported	5	0.5
Marital status		
Single	478	47.3
Married	442	43.7
Divorced	63	6.2
Widowed	22	2.2
Not reported	6	0.6
Education		
Literate without formal schooling	11	1.1
Primary school	76	7.5
Middle school	96	9.5
High school	225	22.3
Undergraduate student	191	18.9
University graduate	317	31.4
Graduate student	43	4.3
Postgraduate degree	48	4.7
Not reported	4	0.4
Perceived income		
Very low	168	16.6
Low	210	20.8
Moderate	446	44.1
High	140	13.8
Very high	42	4.2
Not reported	5	0.5
Place of residence		
Metropolitan city	605	59.8
City	202	20.0
District	147	14.5
Village	51	5.0
Not reported	6	0.6
Lifetime psychiatric diagnosis		
Yes	225	22.3
No	773	76.5
Not reported	13	1.3
Psychotherapy history		
Yes	109	10.8
No	896	88.6
Not reported	6	0.6
Psychiatric medication use		
Yes	214	21.2
No	786	77.7
Not reported	11	1.1

Note. Age ranged from 18 to 65 years (*M* = 33.44, *SD* = 11.61).

**Table 2:** Descriptive statistics of the Turkish MEDI subscales (*N* = 1011).

Subscale	Items	*M*	*SD*	Skewness	Kurtosis
Neurotic Temperament	5	19.46	9.89	0.07	−0.84
Positive Temperament	5	25.47	8.83	−0.60	−0.28
Depressed Mood	5	16.21	9.72	0.32	−0.63
Autonomic Arousal	5	13.99	10.64	0.47	−0.79
Somatic Anxiety	5	15.89	9.43	0.43	−0.52
Social Anxiety	5	14.33	9.89	0.50	−0.51
Intrusive Cognitions	6	16.94	12.14	0.51	−0.60
Traumatic Re-experiencing	5	15.89	10.06	0.40	−0.64
Avoidance	8	27.50	13.51	0.30	−0.38

Note. Items are rated from 0 to 8; higher scores indicate higher levels of the dimension.

**Table 3:** Standardized loadings from the exploratory ESEM (MLR, quartimin) in the first half-sample (*n* = 505).

Item	NT	PT	DM	AA	SOM	SOC	IC	AVD	TRM
Item 1	**0.70**	−0.03	0.08	0.05	−0.02	−0.04	0.10	0.01	−0.02
Item 10	**0.61**	−0.16	0.10	0.07	0.03	0.05	−0.02	0.02	0.11
Item 16	**0.67**	−0.04	0.04	0.04	0.02	0.11	0.15	0.06	−0.04
Item 32	**0.53**	−0.12	0.05	−0.01	0.16	0.16	0.10	0.01	0.03
Item 35	**0.53**	0.02	0.08	−0.19	0.08	−0.03	−0.05	0.18	0.07
Item 2	0.03	**0.65**	0.10	−0.06	−0.01	0.03	0.00	−0.06	0.02
Item 17	−0.11	**0.67**	−0.05	−0.08	0.03	−0.03	0.00	0.06	−0.01
Item 24	−0.02	**0.83**	−0.10	0.14	−0.07	−0.01	0.04	0.02	−0.10
Item 33	−0.19	**0.57**	0.11	−0.07	0.03	−0.13	0.05	0.01	0.08
Item 36	0.08	**0.50**	0.03	−0.38	0.05	−0.06	−0.15	0.16	0.07
Item 3	0.26	0.10	**0.41**	0.00	−0.04	0.08	0.17	0.01	−0.02
Item 11	0.08	−0.21	**0.49**	0.00	−0.06	0.09	0.10	0.08	0.10
Item 25	0.10	0.00	**0.64**	−0.03	−0.02	0.11	0.04	0.05	0.02
Item 37	0.10	−0.03	**0.54**	−0.06	0.04	0.06	0.17	0.07	−0.05
Item 43	−0.05	−0.18	**0.41**	0.12	0.05	0.10	0.19	0.16	0.02
Item 4	0.21	0.10	0.22	**0.25**	0.32	−0.01	−0.12	−0.11	0.20
Item 13	0.10	0.03	0.25	**0.37**	0.28	0.14	−0.04	−0.11	0.15
Item 18	0.21	0.01	0.14	**0.39**	0.23	0.13	−0.02	−0.19	0.20
Item 26	−0.01	−0.03	0.23	**0.19**	0.36	0.12	0.03	−0.03	0.13
Item 44	0.01	0.04	0.16	**0.11**	0.42	0.21	0.00	−0.01	0.16
Item 6	0.39	0.02	0.01	0.04	**0.34**	−0.04	0.05	−0.03	0.12
Item 19	0.23	0.01	−0.12	−0.05	**0.56**	0.06	0.13	0.04	0.03
Item 28	0.06	−0.13	0.01	0.09	**0.46**	0.00	0.34	0.03	0.05
Item 38	−0.01	−0.01	−0.11	−0.04	**0.47**	0.08	0.02	0.32	−0.12
Item 45	−0.05	−0.16	0.08	−0.04	**0.55**	0.06	0.23	0.01	0.05
Item 7	0.03	−0.05	0.10	0.13	−0.02	**0.59**	0.04	0.00	−0.07
Item 14	0.06	0.13	0.11	−0.02	−0.08	**0.60**	0.04	−0.02	0.09
Item 22	0.16	0.13	−0.15	0.01	0.02	**0.71**	0.02	0.04	0.05
Item 41	−0.07	−0.06	0.07	0.01	0.06	**0.76**	0.02	−0.01	0.03
Item 47	−0.08	−0.10	0.02	−0.06	0.01	**0.84**	0.00	0.02	−0.01
Item 5	0.03	0.05	0.23	0.06	−0.07	0.07	**0.44**	−0.04	0.02
Item 12	0.18	0.02	0.11	−0.11	−0.02	0.02	**0.55**	0.03	0.15
Item 21	0.14	0.09	−0.05	0.02	0.03	0.11	**0.69**	−0.03	0.07
Item 30	0.00	−0.14	0.07	0.12	0.11	0.05	**0.54**	0.02	0.09
Item 40	0.04	−0.01	0.08	−0.03	0.13	0.01	**0.60**	−0.01	0.16
Item 46	−0.07	−0.02	0.17	0.08	0.09	0.05	**0.60**	−0.05	0.08
Item 9	0.11	0.05	0.10	0.11	−0.10	−0.01	−0.03	**0.51**	0.07
Item 15	0.05	0.08	−0.02	−0.05	−0.09	0.01	−0.06	**0.65**	0.03
Item 23	0.05	−0.07	−0.08	0.55	0.01	0.05	0.19	**0.27**	0.03
Item 27	−0.01	−0.04	0.02	0.18	0.02	0.03	0.09	**0.49**	0.23
Item 31	−0.02	0.05	0.04	−0.02	0.12	−0.01	−0.04	**0.76**	0.02
Item 34	0.24	0.00	0.09	−0.04	0.01	0.15	−0.09	**0.49**	0.08
Item 42	−0.07	−0.11	0.10	0.24	0.01	0.08	0.13	**0.44**	0.06
Item 49	0.03	−0.13	0.06	0.34	−0.09	0.12	0.12	**0.29**	0.27
Item 8	0.22	−0.04	0.01	−0.07	−0.11	0.04	0.02	0.01	**0.63**
Item 20	−0.06	−0.01	−0.03	0.12	0.05	0.12	0.06	−0.04	**0.66**
Item 29	−0.06	−0.05	0.03	−0.07	0.05	−0.01	0.12	0.09	**0.71**
Item 39	−0.06	−0.04	0.06	0.10	0.06	−0.01	0.05	−0.01	**0.74**
Item 48	0.06	0.03	−0.07	−0.11	−0.04	0.09	0.05	0.16	**0.65**

Note. *N* = 505 (exploratory half-sample). Exploratory structural equation model estimated with robust maximum likelihood and quartimin rotation, following Rosellini and Brown (2019); the model specifies a fully saturated loading matrix with no correlated residuals and is therefore equivalent to an exploratory factor analysis. Target (a priori) loadings are in boldface. Model fit (robust): χ^2^(771) = 1555.40, CFI = 0.95, TLI = 0.93, RMSEA = 0.046 [0.042, 0.050], SRMR = 0.024. Item numbers follow the original MEDI numbering. NT = neurotic temperament; PT = positive temperament; DM = depressed mood; AA = autonomic arousal; SOM = somatic anxiety; SOC = social anxiety; IC = intrusive cognitions; AVD = avoidance; TRM = traumatic re-experiencing.

**Table 4:** Standardized loadings and reliability from the confirmatory ESEM (MLR, quartimin) in the second half-sample (*n* = 506).

Item	NT	PT	DM	AA	SOM	SOC	IC	AVD	TRM	α/ω
Item 1	**0.48**	0.02	0.08	0.20	−0.01	0.02	0.26	0.11	−0.06	0.85/0.85
Item 10	**0.45**	−0.23	0.08	−0.01	0.11	0.11	0.09	0.02	0.21	
Item 16	**0.56**	−0.14	0.04	0.06	0.09	0.12	0.08	0.10	0.08	
Item 32	**0.34**	−0.24	0.07	0.06	0.05	0.18	0.28	0.10	0.02	
Item 35	**0.46**	0.04	0.04	0.18	0.06	0.03	0.02	0.12	0.02	
Item 2	−0.01	**0.64**	0.01	0.04	−0.04	0.05	0.10	0.07	−0.01	0.81/0.82
Item 17	−0.05	**0.71**	0.03	−0.02	0.08	−0.10	−0.14	−0.05	0.11	
Item 24	−0.08	**0.76**	−0.12	0.07	−0.05	−0.03	0.09	0.00	−0.09	
Item 33	−0.22	**0.52**	0.02	−0.08	0.05	−0.03	−0.01	0.05	0.05	
Item 36	0.15	**0.64**	0.00	−0.13	0.04	−0.01	−0.05	0.09	0.00	
Item 3	0.21	0.14	**0.51**	0.05	−0.07	0.07	0.07	0.00	0.05	0.81/0.82
Item 11	0.09	−0.22	**0.43**	0.06	0.11	0.07	0.07	0.07	0.03	
Item 25	0.00	0.00	**0.71**	0.06	−0.04	0.01	0.04	−0.01	−0.04	
Item 37	−0.07	−0.01	**0.72**	−0.03	0.05	0.06	−0.01	0.04	0.04	
Item 43	−0.17	−0.18	**0.43**	0.01	0.12	0.07	0.07	−0.02	0.06	
Item 4	0.09	0.09	0.09	**0.69**	−0.04	0.04	−0.02	0.06	0.05	0.87/0.87
Item 13	−0.02	−0.08	−0.02	**0.57**	0.06	0.07	0.04	−0.01	0.21	
Item 18	0.12	−0.05	−0.06	**0.52**	0.11	0.04	0.13	0.07	0.11	
Item 26	−0.06	−0.02	0.07	**0.70**	0.10	0.07	0.03	0.01	0.00	
Item 44	0.10	−0.05	0.08	**0.57**	0.11	0.00	0.04	0.01	0.02	
Item 6	0.13	0.09	0.18	0.16	**0.47**	0.03	0.00	0.06	0.04	0.80/0.80
Item 19	0.15	0.11	0.01	0.12	**0.64**	−0.04	0.04	0.02	0.03	
Item 28	−0.09	−0.13	0.09	0.21	**0.53**	0.07	0.16	−0.12	0.06	
Item 38	−0.06	0.03	−0.07	−0.16	**0.63**	0.10	−0.02	0.15	−0.07	
Item 45	−0.04	−0.08	0.14	0.23	**0.39**	0.14	0.12	−0.17	−0.02	
Item 7	−0.02	−0.02	0.04	−0.03	−0.02	**0.67**	0.01	0.00	0.07	0.86/0.86
Item 14	0.01	0.00	−0.01	0.00	0.03	**0.62**	−0.03	0.04	0.00	
Item 22	0.16	0.20	−0.03	0.03	0.07	**0.65**	0.01	0.01	0.09	
Item 41	−0.04	−0.07	−0.04	0.09	0.04	**0.74**	0.03	0.03	−0.01	
Item 47	−0.01	−0.04	0.08	−0.03	−0.03	**0.90**	−0.03	−0.03	−0.02	
Item 5	0.04	0.16	0.21	−0.01	−0.01	0.12	**0.41**	0.04	−0.05	0.88/0.88
Item 12	0.23	0.03	0.10	−0.07	0.02	−0.08	**0.62**	0.05	0.13	
Item 21	0.13	0.12	0.01	0.07	−0.01	0.07	**0.70**	−0.03	0.08	
Item 30	−0.11	−0.13	0.01	0.03	0.17	0.06	**0.65**	0.05	0.00	
Item 40	−0.06	−0.09	0.13	0.02	0.04	−0.10	**0.58**	0.02	0.23	
Item 46	−0.08	−0.03	0.06	0.09	0.05	0.10	**0.62**	0.06	−0.03	
Item 9	0.07	0.08	0.04	−0.01	0.03	0.02	0.04	**0.61**	−0.08	0.83/0.83
Item 15	0.12	0.15	0.01	−0.09	−0.03	−0.01	−0.04	**0.63**	−0.06	
Item 23	−0.28	−0.21	−0.06	0.15	0.02	0.14	0.21	**0.35**	0.07	
Item 27	−0.19	−0.05	0.00	0.13	0.04	0.05	0.10	**0.56**	0.12	
Item 31	0.01	0.04	−0.04	−0.03	0.03	0.02	−0.04	**0.67**	0.04	
Item 34	0.18	−0.01	0.06	0.04	0.00	0.00	−0.01	**0.64**	0.07	
Item 42	−0.26	−0.12	0.08	0.17	0.08	0.03	0.10	**0.50**	0.06	
Item 49	−0.09	−0.18	0.17	0.08	0.05	0.10	0.14	**0.35**	0.14	
Item 8	0.10	0.03	0.00	−0.19	0.10	0.11	0.04	0.03	**0.61**	0.85/0.86
Item 20	−0.04	0.06	−0.07	0.23	−0.04	0.16	0.09	−0.08	**0.56**	
Item 29	−0.07	−0.02	0.01	0.02	0.07	0.00	0.12	0.09	**0.68**	
Item 39	−0.02	−0.01	0.13	0.13	−0.07	0.00	0.03	−0.04	**0.73**	
Item 48	0.16	0.02	−0.01	0.06	−0.01	0.04	−0.08	0.22	**0.54**	

Note. *N* = 506 (confirmatory half-sample). Model fit (robust): χ^2^(771) = 1532.60, CFI = 0.95, TLI = 0.92, RMSEA = 0.046 [0.041, 0.050], SRMR = 0.024. Target (a priori) loadings are in boldface. α = Cronbach’s alpha; ω = McDonald’s omega (each computed from a single-factor model of the subscale’s items) in the full sample (*N* = 1011). NT = neurotic temperament; PT = positive temperament; DM = depressed mood; AA = autonomic arousal; SOM = somatic anxiety; SOC = social anxiety; IC = intrusive cognitions; AVD = avoidance; TRM = traumatic re-experiencing.

**Table 5:** Measurement invariance of the nine-factor model across administration mode (online vs. paper-and-pencil). ESEM = exploratory structural equation modeling (MLR, robust fit indices); CFA = confirmatory factor analysis (ML).

Model	χ^2^ (*df*)	CFI	RMSEA [90% CI]	SRMR	ΔCFI	ΔRMSEA	ΔSRMR
ESEM configural	3478.58 (1542)	0.941	0.050 [0.047, 0.053]	0.025	—	—	—
ESEM metric	3984.27 (1902)	0.920	0.049 [0.047, 0.051]	0.039	−0.021	−0.001	0.014
ESEM scalar	4034.12 (1942)	0.919	0.049 [0.046, 0.051]	0.039	−0.001	0.000	0.000
CFA configural	7034.83 (2182)	0.821	0.066 [0.065, 0.068]	0.072	—	—	—
CFA metric	7135.71 (2222)	0.819	0.066 [0.064, 0.068]	0.074	−0.002	0.000	0.002
CFA scalar	7327.76 (2262)	0.813	0.067 [0.065, 0.068]	0.074	−0.006	0.000	0.000

Note. *df* = degrees of freedom; CI = confidence interval; CFI = comparative fit index; RMSEA = root mean square error of approximation; SRMR = standardized root mean square residual; Δ = change relative to the preceding, less constrained model. Invariance was evaluated against ΔCFI ≤ 0.010, ΔRMSEA ≤ 0.015, and ΔSRMR ≤ 0.030 (metric)/≤0.010 (scalar) (Chen, 2007).

**Table 6:** Measurement invariance of the nine-factor model across gender (female vs. male; *n* = 1006). ESEM = exploratory structural equation modeling (MLR, robust fit indices); CFA = confirmatory factor analysis (ML).

Model	χ^2^ (*df*)	CFI	RMSEA [90% CI]	SRMR	ΔCFI	ΔRMSEA	ΔSRMR
ESEM configural	3173.13 (1542)	0.949	0.047 [0.044, 0.049]	0.024	—	—	—
ESEM metric	3483.79 (1902)	0.938	0.043 [0.041, 0.046]	0.037	−0.011	−0.004	0.013
ESEM scalar	3534.20 (1942)	0.938	0.043 [0.040, 0.045]	0.037	0.000	0.000	0.000
CFA configural	6580.84 (2182)	0.835	0.063 [0.062, 0.065]	0.067	—	—	—
CFA metric	6648.77 (2222)	0.834	0.063 [0.061, 0.065]	0.070	−0.001	0.000	0.003
CFA scalar	6754.58 (2262)	0.832	0.063 [0.061, 0.065]	0.071	−0.002	0.000	0.000

Note. *df* = degrees of freedom; CI = confidence interval; CFI = comparative fit index; RMSEA = root mean square error of approximation; SRMR = standardized root mean square residual; Δ = change relative to the preceding, less constrained model. Invariance was evaluated against ΔCFI ≤ 0.010, ΔRMSEA ≤ 0.015, and ΔSRMR ≤ 0.030 (metric)/≤0.010 (scalar) (Chen, 2007).

**Table 7:** Pearson correlations between the MEDI subscales and the criterion measures.

MEDI Subscale	BFI-N	BFI-E	DASS-D	DASS-A	BFNE	OCI-R Obs	MEAQ	ASI-3 Phys	PCL-5 Re-exp
Neurotic Temperament	**0.86**	—	—	—	—	—	—	—	—
Positive Temperament	−0.21	**0.83**	—	—	—	—	—	—	—
Depressed Mood	0.50	−0.27	**0.75**	—	—	—	—	—	—
Autonomic Arousal	0.50	−0.24	0.42	**0.81**	—	—	—	—	—
Social Anxiety	0.46	−0.23	0.38	0.44	**0.58**	—	—	—	—
Intrusive Cognitions	0.50	−0.23	0.50	0.51	0.32	**0.79**	—	—	—
Avoidance	0.41	−0.12	0.33	0.33	0.30	0.39	**0.81**	—	—
Somatic Anxiety	0.44	−0.22	0.37	0.52	0.31	0.48	0.30	**0.54**	—
Traumatic Re-experiencing	0.45	−0.12	0.38	0.47	0.32	0.48	0.42	0.23	**0.83**

Note. *N* = 1011. BFI-N/BFI-E = Big Five Inventory Neuroticism/Extraversion; DASS-D/DASS-A = Depression Anxiety Stress Scales-21 Depression/Anxiety; BFNE = Brief Fear of Negative Evaluation Scale; OCI-R Obs = Obsessive-Compulsive Inventory-Revised Obsessing subscale; MEAQ = Multidimensional Experiential Avoidance Questionnaire; ASI-3 Phys = Anxiety Sensitivity Index-3 Physical Concerns subscale; PCL-5 Re-exp = PTSD Checklist for DSM-5 Re-experiencing subscale. Boldface values denote the a priori convergent validity pairs. All correlations are significant at *p* < 0.001.

**Table 8:** Gender-specific normative data (percentile ranks and *T*-scores) for the nine MEDI dimensions.

MEDI Dimension	Women (*n* = 574)			Men (*n* = 432)		
	P25	P50	P75	P25	P50	P75
Neurotic Temperament	12 (42)	20 (50)	27 (58)	11 (42)	19 (50)	27 (57)
Positive Temperament	21 (44)	28 (52)	33 (58)	18 (43)	26 (52)	32 (58)
Depressed Mood	7 (41)	15 (50)	22 (57)	10 (42)	17 (50)	24 (57)
Autonomic Arousal	5 (42)	13 (49)	21 (57)	4 (41)	13 (49)	23 (58)
Social Anxiety	6 (42)	13 (49)	20 (56)	6 (41)	14 (49)	21 (56)
Intrusive Cognitions	6 (41)	14 (48)	24 (57)	7 (41)	16 (48)	27 (57)
Avoidance	18 (43)	27 (49)	37 (57)	17 (43)	26 (50)	37 (57)
Somatic Anxiety	9 (43)	14 (48)	21 (56)	9 (42)	15 (48)	24 (57)
Traumatic Re-experiencing	8 (42)	16 (50)	23 (57)	7 (42)	15 (50)	23 (57)

Note. Values are raw scores with the corresponding *T*-score in parentheses at the 25th (P25), 50th (P50), and 75th (P75) percentiles. *T*-scores were computed within each gender group (*M* = 50, *SD* = 10). Five participants who did not report their gender were excluded.

## Data Availability

The data presented in this study are available on request from the corresponding author due to ethical and privacy restrictions.

## Supplementary Materials

The following supporting information can be downloaded at: https://www.mdpi.com/article/10.3390/bs16101957/s1, Table S1: Sociodemographic comparison of the exploratory and confirmatory half-samples, Table S2: Item-level response distributions of the Turkish MEDI, Table S3: Equivalence of the English and Turkish forms of the MEDI in the bilingual sample (*N* = 45). References (Lakens, 2017; McGraw & Wong, 1996) are cited in the Supplementary Materials.

## Abbreviations

The following abbreviations are used in this manuscript:

MEDI	Multidimensional Emotional Disorder Inventory
ED	Emotional disorder
ESEM	Exploratory structural equation modeling
CFA	Confirmatory factor analysis
EFA	Exploratory factor analysis
MLR	Robust maximum likelihood
FIML	Full-information maximum likelihood
CFI	Comparative fit index
TLI	Tucker–Lewis index
RMSEA	Root mean square error of approximation
SRMR	Standardized root mean square residual
BFI	Big Five Inventory
DASS-21	Depression Anxiety Stress Scales-21
BFNE	Brief Fear of Negative Evaluation Scale
OCI-R	Obsessive-Compulsive Inventory-Revised
ASI-3	Anxiety Sensitivity Index-3
PCL-5	PTSD Checklist for DSM-5
MEAQ	Multidimensional Experiential Avoidance Questionnaire
UP	Unified Protocol
