# Supplementary material for: Multidimensional Emotional Disorder Inventory: Reliability and Validity in a Turkish Community Sample
Source: Behav Sci (Basel). 2026 Oct 21;16(10):1957. doi: 10.3390/bs16101957 (PMC13701914; doi:10.3390/bs16101957)
Supplement: Supplementary file 1 [file behavsci-16-01957-s001.zip › behavsci-4623525-supplementary.pdf]

Supplementary Materials

Multidimensional Emotional Disorder Inventory: Reliability and Validity in a Turkish Community Sample

Table S1. Sociodemographic comparison of the exploratory and confirmatory half-samples.

Characteristic	Exploratory half-sample (n = 505)	Confirmatory half-sample (n = 506)	Test	p	Effect size
Age, M (SD)	33.54 (11.39)	33.34 (11.84)	t(998) = 0.27	.788	d = .02
**Gender**			**$\chi^2(1) = 1.62$**	**.203**	**V = .04**
Female	277 (55.1)	297 (59.0)			
Male	226 (44.9)	206 (41.0)			
**Marital status**			**$\chi^2(3) = 5.27$**	**.153**	**V = .07**
Single	233 (46.4)	245 (48.7)			
Married	225 (44.8)	217 (43.1)			
Widowed	7 (1.4)	15 (3.0)			
Divorced	37 (7.4)	26 (5.2)			
**Education**			**$\chi^2(7) = 6.46$**	**.487**	**V = .08**
Literate, no formal schooling	4 (0.8)	7 (1.4)			
Primary school	41 (8.2)	35 (6.9)			
Middle school	41 (8.2)	55 (10.9)			
High school	112 (22.3)	113 (22.4)			
University student	101 (20.1)	90 (17.8)			
University graduate	152 (30.3)	165 (32.7)			
Graduate student	26 (5.2)	17 (3.4)			
Graduate degree	25 (5.0)	23 (4.6)			
**Income level**			**$\chi^2(4) = 1.01$**	**.908**	**V = .03**
Very low	87 (17.3)	81 (16.1)			
Low	103 (20.5)	107 (21.3)			
Middle	218 (43.3)	228 (45.3)			
High	72 (14.3)	68 (13.5)			
Very high	23 (4.6)	19 (3.8)			
**Place of residence**			**$\chi^2(3) = 10.05$**	**.018**	**V = .10**
Metropolitan city	287 (57.1)	318 (63.3)			
City	120 (23.9)	82 (16.3)			
District	68 (13.5)	79 (15.7)			
Village	28 (5.6)	23 (4.6)			

Note. Values are n (%) unless otherwise indicated. Percentages are based on participants with valid data. The total sample was randomly split into two half-samples, stratified by administration mode, so that each half-sample contained 295 online participants and 210 or 211 paper-and-pencil participants. Categorical variables were compared with χ^2 tests (effect size: Cramér's V), and age was compared with a Welch t test (effect size: Cohen's d).

Table S2. Item-level response distributions of the Turkish MEDI (N = 1,011).

Item	Dimension	0	1	2	3	4	5	6	7	8	M	SD	Skew	Kurt	n
1	NT	13.4	8.0	9.8	9.8	18.4	10.8	11.5	8.2	10.2	3.92	2.51	−0.02	−1.05	1011
2	PT	6.5	5.9	9.5	8.5	17.8	12.0	15.3	10.6	13.8	4.58	2.37	−0.27	−0.88	1011
3	DM	19.8	8.0	11.1	12.2	11.6	9.6	10.5	6.3	11.0	3.56	2.67	0.17	−1.18	1011
4	AA	22.0	15.3	8.6	6.3	14.8	9.7	10.2	6.8	6.2	3.18	2.60	0.28	−1.20	1011
5	IC	23.6	12.6	12.3	12.0	10.6	9.4	8.1	5.1	6.3	2.98	2.53	0.45	−0.96	1011
6	SOM	14.1	12.4	10.7	9.7	15.5	11.1	10.0	7.9	8.6	3.64	2.53	0.13	−1.12	1011
7	SOC	31.6	12.8	11.2	8.6	13.6	7.3	6.0	3.3	5.7	2.57	2.49	0.65	−0.69	1011
8	TRM	14.0	9.9	12.4	10.0	17.1	10.6	9.5	6.6	9.9	3.68	2.51	0.14	−1.05	1011
9	AVD	12.9	7.9	8.7	8.0	17.2	12.8	12.3	9.0	11.3	4.09	2.54	−0.13	−1.07	1011
10	NT	14.7	11.0	10.5	10.8	16.8	9.3	8.9	8.7	9.3	3.67	2.55	0.14	−1.11	1011
11	DM	16.0	12.5	13.9	11.8	12.8	9.4	9.9	5.7	8.0	3.37	2.50	0.30	−1.03	1010
12	IC	18.2	11.8	14.1	10.4	11.5	10.6	8.0	6.1	9.3	3.35	2.59	0.32	−1.08	1011
13	AA	31.4	14.0	9.8	5.9	12.7	9.1	5.8	5.6	5.6	2.67	2.59	0.58	−0.94	1011
14	SOC	21.5	13.2	11.1	9.9	16.7	10.0	7.7	3.6	6.3	3.04	2.45	0.38	−0.89	1009
15	AVD	9.6	7.0	8.2	10.9	15.5	11.9	12.8	10.3	13.8	4.37	2.50	−0.19	−1.02	1011
16	NT	13.6	9.8	12.1	8.8	18.1	10.9	10.3	6.7	9.8	3.74	2.50	0.10	−1.05	1011
17	PT	4.9	6.4	5.2	8.3	13.6	10.9	14.4	13.5	22.7	5.13	2.43	−0.52	−0.79	1011
18	AA	37.4	12.7	10.5	7.7	9.1	8.2	4.8	4.3	5.3	2.36	2.54	0.79	−0.61	1011
19	SOM	18.5	10.6	11.5	11.7	13.7	10.5	9.6	6.1	7.8	3.39	2.53	0.23	−1.07	1011
20	TRM	30.7	15.5	10.6	7.9	11.9	6.5	8.1	3.3	5.4	2.56	2.51	0.67	−0.75	1010
21	IC	25.3	14.0	11.5	7.6	12.7	9.3	7.4	5.0	7.1	2.94	2.59	0.47	−1.00	1010
22	SOC	17.1	12.9	11.7	10.5	16.8	9.7	8.8	4.9	7.5	3.31	2.47	0.29	−0.96	1009
23	AVD	41.9	12.3	9.8	5.1	9.6	7.1	5.1	3.7	5.5	2.21	2.56	0.89	−0.49	1009
24	PT	5.8	4.3	5.4	6.2	17.0	15.9	16.5	13.9	14.8	4.97	2.26	−0.56	−0.44	1011
25	DM	18.6	9.9	10.5	14.2	14.4	10.8	7.4	6.1	8.0	3.37	2.50	0.26	−0.98	1011
26	AA	31.0	12.8	7.4	6.2	14.8	7.8	7.2	6.0	6.7	2.84	2.67	0.47	−1.09	1011
27	AVD	17.2	11.6	10.5	10.0	18.0	12.0	8.2	4.0	8.5	3.40	2.48	0.23	−0.95	1010
28	SOM	33.7	15.5	10.8	8.5	8.1	7.5	6.3	3.4	6.1	2.43	2.54	0.79	−0.60	1011
29	TRM	24.4	13.3	9.4	8.3	16.0	8.2	7.0	4.2	9.2	3.07	2.63	0.42	−1.01	1010
30	IC	37.3	13.5	9.5	5.6	12.9	7.8	4.8	3.4	5.2	2.34	2.51	0.78	−0.59	1010
31	AVD	9.0	8.8	10.8	11.1	14.9	13.7	12.2	9.0	10.5	4.12	2.42	−0.07	−1.02	1011
32	NT	18.7	11.5	11.2	7.4	16.5	11.6	9.3	6.5	7.3	3.40	2.54	0.19	−1.11	1011
33	PT	4.8	6.5	7.7	7.4	19.2	15.4	12.9	11.5	14.5	4.72	2.29	−0.31	−0.76	1011
34	AVD	10.5	11.1	10.1	9.8	15.2	11.6	12.3	7.2	12.3	4.02	2.53	−0.00	−1.12	1011
35	NT	7.4	5.5	6.7	8.3	17.2	12.9	13.8	12.0	16.1	4.73	2.42	−0.37	−0.82	1011

Item	Dimension	0	1	2	3	4	5	6	7	8	M	SD	Skew	Kurt	n
36	PT	3.7	3.4	3.1	2.9	12.5	8.0	10.4	13.7	42.4	6.06	2.31	−1.09	0.22	1011
37	DM	18.0	10.8	10.7	12.7	14.0	10.1	8.1	7.3	8.3	3.43	2.55	0.24	−1.07	1010
38	SOM	12.5	7.7	11.0	8.4	22.8	12.7	11.8	5.6	7.5	3.80	2.34	−0.02	−0.86	1011
39	TRM	26.6	14.5	13.3	9.5	12.2	8.2	6.4	2.9	6.4	2.70	2.47	0.63	−0.68	1010
40	IC	29.6	11.4	10.6	6.0	14.0	9.1	7.7	4.5	7.0	2.87	2.63	0.47	−1.04	1011
41	SOC	24.9	16.0	12.9	8.4	14.8	7.4	6.6	3.8	5.1	2.71	2.42	0.59	−0.72	1011
42	AVD	27.0	12.5	12.1	9.1	13.9	8.2	7.6	3.5	6.1	2.80	2.50	0.52	−0.85	1011
43	DM	37.1	10.6	10.5	8.4	9.6	7.1	6.2	4.4	6.0	2.47	2.60	0.72	−0.75	1010
44	AA	29.8	13.6	10.0	5.6	8.9	8.6	8.8	6.1	8.6	2.94	2.77	0.47	−1.19	1011
45	SOM	38.3	10.4	7.4	6.5	8.4	8.7	7.7	4.8	7.6	2.63	2.76	0.61	−1.05	1008
46	IC	35.5	12.8	11.6	5.8	10.0	9.5	4.8	4.1	5.9	2.46	2.57	0.73	−0.71	1011
47	SOC	27.8	14.0	12.2	7.0	14.3	9.1	5.0	4.1	6.4	2.72	2.52	0.59	−0.78	1011
48	TRM	11.9	12.3	9.3	8.4	17.5	11.6	11.1	6.8	11.1	3.87	2.53	0.04	−1.11	1010
49	AVD	32.8	14.6	11.0	8.2	8.3	9.7	4.7	4.9	5.6	2.51	2.55	0.72	−0.73	1011

Note. Columns 0–8 give the percentage of respondents selecting each response option (0 = not characteristic of me/does not apply to me; 8 = extremely characteristic of me/applies to me very much). Item numbers follow the original MEDI numbering. Percentages and descriptive statistics are based on observed responses, so n varies slightly because of missing responses. NT = neurotic temperament; PT = positive temperament; DM = depressed mood; AA = autonomic arousal; SOM = somatic anxiety; SOC = social anxiety; IC = intrusive cognitions; AVD = avoidance; TRM = traumatic re-experiencing.

Table S3. Equivalence of the English and Turkish forms of the MEDI in the bilingual sample (N = 45).

Subscale	English M (SD)	Turkish M (SD)	Mean difference	t(44) (p)	r	90% CI of difference	Equivalence bounds	pTOST	ICC(A,1) [95% CI]
Neurotic Temperament	21.69 (10.70)	21.22 (10.80)	0.47	1.23 (.227)	.97	[−0.17, 1.11]	±1.28	.019	.97 [.95, .98]
Positive Temperament	26.27 (6.69)	25.62 (6.60)	0.64	1.15 (.255)	.84	[−0.29, 1.58]	±1.87	.017	.84 [.73, .91]
Depressed Mood	17.07 (10.63)	17.49 (10.49)	−0.42	−1.21 (.233)	.98	[−1.01, 0.16]	±1.17	.019	.98 [.96, .99]
Autonomic Arousal	17.13 (10.78)	16.60 (11.04)	0.53	1.14 (.260)	.96	[−0.25, 1.32]	±1.57	.016	.96 [.93, .98]
Somatic Anxiety	18.40 (8.94)	17.93 (9.28)	0.47	1.27 (.211)	.96	[−0.15, 1.08]	±1.23	.022	.96 [.93, .98]
Social Anxiety	17.71 (11.43)	17.76 (11.26)	−0.04	−0.15 (.881)	.98	[−0.54, 0.45]	±0.99	.001	.99 [.97, .99]
Intrusive Cognitions	23.42 (11.36)	23.24 (11.16)	0.18	0.29 (.770)	.94	[−0.84, 1.19]	±2.02	.002	.94 [.89, .96]
Traumatic Re-experiencing	18.87 (10.31)	18.73 (10.42)	0.13	0.28 (.778)	.95	[−0.66, 0.92]	±1.58	.002	.95 [.92, .97]
Avoidance	30.31 (12.40)	30.98 (12.64)	−0.67	−0.90 (.373)	.92	[−1.91, 0.58]	±2.49	.009	.92 [.86, .96]

Note. Mean difference = English minus Turkish subscale score. Equivalence was tested with two one-sided tests (TOST; Lakens, 2017) against bounds of ±0.5 standard deviations of the difference scores (shown in raw score units). pTOST is the larger of the two one-sided p values, and equivalence is supported when pTOST < .05 (equivalently, when the 90% CI of the difference lies within the bounds). ICC(A,1) = two-way random-effects intraclass correlation coefficient for absolute agreement, single measures (McGraw & Wong, 1996).
